# Our computational nature: comment on Barrett et al.

**DOI:** 10.3389/fpsyg.2014.01348

**Published:** 2014-11-21

**Authors:** John Klasios

**Affiliations:** York UniversityToronto, ON, Canada

**Keywords:** evolutionary psychology, computationalism, human nature, computers, cognitive science

I argue that Barrett et al. (2014) have misinterpreted evolutionary psychologists' notion of computation. Barrett et al. seemingly presume that the notion of computation deployed by evolutionary psychologists (e.g., Pinker, 1997; Tooby and Cosmides, 2005) is tantamount to positing a *physical* architecture whose form of computation proceeds via the syntactic-like transformations of spatially discrete representational symbols or sentence-like structures—i.e., in the manner of a Turing machine. But this is simply not the notion of computation that evolutionary psychologists advocate (in spite of the fact that a Turing machine architecture, for instance, is nonetheless compatible with it).

The notion of computation is philosophically complex, with many different meanings and a multifaceted history (Piccinini, 2012). By “computation,” evolutionary psychologists fundamentally mean to say that the brain evolved to compute in the generic sense of the term. This more generic notion of computation, and its relation to a physical substrate, is outlined by Pinker (2005):

“Computation” … does not refer to what a commercially available digital computer does but to a more generic notion of mechanical rationality …. In this conception, a computational system is one in which knowledge and goals are represented as *patterns* in bits of matter (“representations”). (p. 2, emphasis added)

The misunderstanding of what evolutionary psychologists mean by computation also leads Barrett et al. to view various other conceptions of cognition—i.e., embodied, embedded, extended, enactive—as alternatives to the computational approach when, in actuality, they can easily be seen as complementary to it. For evolutionary psychologists are primarily focused on the functional level of analysis of psychological adaptations rather than their physical instantiations (i.e., their causal–physical basis in the brain, body, and wider environment). And this focus on the functional level of analysis allows researchers to investigate psychological adaptations in a manner that abstracts away from their instantiations in the brain, body, and larger context (i.e., ecological) in which they are embedded. The modular, computational framework of evolutionary psychology is quite compatible with, and can be meaningfully situated within, an overall physical and causal account that is highly complex, widely distributed, and highly diffuse. So, far from invalidating or highlighting a “prejudice” inherent to computationalism or evolutionary psychology, the supposed alternative approach that Barrett et al. advocate is rather a difference of focus and emphasis. For there is nothing within the theoretical approach of evolutionary psychology that in principle denies the existence of the kinds of “E-cognition” that Barrett et al. draw attention to. At a pragmatic level, different research programs will simply find it profitable to have differing explanatory focuses and emphases.

Barrett et al. also raise skepticism regarding the relationship between psychological adaptations and their neurobiological underpinnings. But it is important to note that the form–function fit that evolutionary psychologists focus on qua adaptationists pertains most directly to aspects of “psychological design” rather than to properties of the neurobiological realization of those designs. Thus, the reverse-engineering approach accordingly homes in on the psychological level of analysis and not the neurobiological one (or at least not primarily). More generally, at this stage of the game it is premature to draw overly strict conclusions on precisely how psychological adaptations may or may not be instantiated in the brain—e.g., if and how they are “multiply realizable” by neurobiological bases, and whether to interpret neuroimaging results according to a regionally-focused or network-wide perspective, etc. (e.g., Klein, 2012; Colombo, 2013).

Barrett and colleagues' discussion of human nature is also problematic. For instance, they endorse Wheeler and Clark's (2008) conception of human nature as “a kind of meta-nature … an extended cognitive architecture whose constancy lies mainly in its continual openness to change” (p. 3572) and lead the reader to believe that it is necessarily at variance with the notion of human nature alluded to by evolutionary psychologists. On the contrary, however, evolutionary psychologists recognize this underlying constancy and refer to it as our underlying “developmental programs” (e.g., Tooby et al., 2003). For evolutionary psychologists, human nature is tantamount to the ontogenesis of our species-typical psychological adaptations. On this view, human nature is envisioned as being expressed through development in ways that are guided, generative, and constrained. The precise details of how guided, generative, and constrained this underlying nature is are of course a complex empirical matter that has barely just commenced, scientifically speaking. At any rate, abstractly modeling postulated psychological adaptations in computational terms is an invaluable method of investigation (see Barrett, 2006, 2007; Bechtel, 2007; Tooby and Cosmides, 2008; Frankenhuis et al., 2013; Levy and Bechtel, 2013).

Indeed, evolutionary psychologists argue that our developmental programs should be computationally mapped in ever-increasing detail, ultimately yielding a high-resolution map of human nature. Barrett et al. claim that human nature can be “cultivated,” shaped, and refracted (etc.). But the extent to which this is possible is ultimately an empirical question, and the more we can understand the nature of our developmental programs, the less need we will have for such vague notions. And in any case, however much we can cultivate our developmental programs, they are fundamentally a product of past selection and thus cannot be adapted—in the *adaptationist* sense—to present and future conditions (e.g., Tooby and Cosmides, 1990).

Barrett et al. also crucially omit certain aspects of evolutionary psychologists' treatment of culture. To wit, Barrett et al. imply that evolutionary psychologists cannot account for the way in which material artifacts, beliefs, and so forth, interact with the underlying developmental programs undergirding our species-typical cognitive architecture. To the contrary: evolutionary psychologists' notion of epidemiological culture refers both to the transmission of culture between individual minds and its impact on the cognitive architecture of those minds (Tooby and Cosmides, 1992; Sperber, 1996). Furthermore, models of epidemiological culture can, in principle, be as complex and dynamic as need be. Hence, the allegations by Barrett et al. that evolutionary psychology is inconsistent with or fails in principle to account for such cultural phenomena are baseless.

## Conflict of interest statement

The author declares that the research was conducted in the absence of any commercial or financial relationships that could be construed as a potential conflict of interest.
